# Telomere-to-telomere and gap-free reference genome assembly of the kiwifruit *Actinidia chinensis*

**DOI:** 10.1093/hr/uhac264

**Published:** 2022-12-02

**Authors:** Junyang Yue, Qinyao Chen, Yingzhen Wang, Lei Zhang, Chen Ye, Xu Wang, Shuo Cao, Yunzhi Lin, Wei Huang, He Xian, Hongyan Qin, Yanli Wang, Sijia Zhang, Ying Wu, Songhu Wang, Yi Yue, Yongsheng Liu

**Affiliations:** School of Horticulture, Anhui Agricultural University, Hefei, Anhui 230036, China; Agricultural Genomics Institute at Shenzhen, Chinese Academy of Agricultural Sciences, Shenzhen, Guangdong 518124, China; School of Horticulture, Anhui Agricultural University, Hefei, Anhui 230036, China; School of Horticulture, Anhui Agricultural University, Hefei, Anhui 230036, China; Institute of Fruit and Tea, Hubei Academy of Agricultural Sciences, Wuhan, Hubei 430064, China; School of Information and Computer, Anhui Agricultural University, Hefei, Anhui 230036, China; Agricultural Genomics Institute at Shenzhen, Chinese Academy of Agricultural Sciences, Shenzhen, Guangdong 518124, China; Agricultural Genomics Institute at Shenzhen, Chinese Academy of Agricultural Sciences, Shenzhen, Guangdong 518124, China; Ministry of Education Key Laboratory for Bio-resource and Eco-environment, College of Life Science, State Key Laboratory of Hydraulic and Mountain River Engineering, Sichuan University, Chengdu, Sichuan 610064, China; Department of Bioinformatics, Anhui Double Helix Gene Technology Corporation, Hefei, Anhui 230022, China; Comprehensive Testing Ground, Xinjiang Academy of Agricultural Sciences, Urumqi, Xinjiang 830012, China; Institute of Special Animal and Plant Sciences, Chinese Academy of Agricultural Sciences, Changchun, Jilin 130112, China; Institute of Special Animal and Plant Sciences, Chinese Academy of Agricultural Sciences, Changchun, Jilin 130112, China; School of Horticulture, Anhui Agricultural University, Hefei, Anhui 230036, China; School of Horticulture, Anhui Agricultural University, Hefei, Anhui 230036, China; School of Horticulture, Anhui Agricultural University, Hefei, Anhui 230036, China; School of Information and Computer, Anhui Agricultural University, Hefei, Anhui 230036, China; School of Horticulture, Anhui Agricultural University, Hefei, Anhui 230036, China; Ministry of Education Key Laboratory for Bio-resource and Eco-environment, College of Life Science, State Key Laboratory of Hydraulic and Mountain River Engineering, Sichuan University, Chengdu, Sichuan 610064, China

## Abstract

Kiwifruit is an economically and nutritionally important fruit crop with extremely high contents of vitamin C. However, the previously released versions of kiwifruit genomes all have a mass of unanchored or missing regions. Here, we report a highly continuous and completely gap-free reference genome of *Actinidia chinensis* cv. ‘Hongyang’, named Hongyang v4.0, which is the first to achieve two *de novo* haploid-resolved haplotypes, HY4P and HY4A. HY4P and HY4A have a total length of 606.1 and 599.6 Mb, respectively, with almost the entire telomeres and centromeres assembled in each haplotype. In comparison with Hongyang v3.0, the integrity and contiguity of Hongyang v4.0 is markedly improved by filling all unclosed gaps and correcting some misoriented regions, resulting in ~38.6–39.5 Mb extra sequences, which might affect 4263 and 4244 protein-coding genes in HY4P and HY4A, respectively. Furthermore, our gap-free genome assembly provides the first clue for inspecting the structure and function of centromeres. Globally, centromeric regions are characterized by higher-order repeats that mainly consist of a 153-bp conserved centromere-specific monomer (*Ach-CEN153*) with different copy numbers among chromosomes. Functional enrichment analysis of the genes located within centromeric regions demonstrates that chromosome centromeres may not only play physical roles for linking a pair of sister chromatids, but also have genetic features for participation in the regulation of cell division. The availability of the telomere-to-telomere and gap-free Hongyang v4.0 reference genome lays a solid foundation not only for illustrating genome structure and functional genomics studies but also for facilitating kiwifruit breeding and improvement.

## Introduction

Kiwifruit, the edible berry of woody vines in the genus *Actinidia*, consists of 54 species and exhibits multiple ploidy levels ranging from di- to octoploid with a base chromosome number of *x* = 29 [1]. It is commonly celebrated as ‘the king of fruits’ thanks to the remarkable high vitamin C content and delicious flavors. Today, hundreds of varieties are cultivated in the world and the total annual sales may exceed 20 billion dollars. Extensive studies on metabolic accumulation and molecular breeding have made great progress since the first draft kiwifruit (*Actinidia chinensis*, NCBI:txid3625, 2*n* = 2*x* = 58) reference genome released over 10 years ago [2]. Further, another five kiwifruit genomes have been successively sequenced so far, including two varieties of *A. chinensis* [3, 4], two varieties of *Actinidia eriantha* [5, 6], and one variety of *Actinidia kolomikta* [7], which have actively promoted the development of kiwifruit functional genomics. However, each genome still contains a large number of unclosed gaps and unanchored sequences, especially in the highly repetitive regions. Besides, the majority of genome assemblies do not contain telomeric and centromeric sequences. For instance, in the latest upgraded reference genome of *A. chinensis* that has been released (namely Hongyang v3.0), 646 unresolved gaps and ~10% unanchored sequences remain in its chimeric monoploid assembly, and the telomeres and centromeres are not mentioned at all[4]. Apparently, the unassembled or unplaced regions are often excluded from genome structural and functional analysis, whichwould seem to limit the utility of these genomes for genomic andmolecular studies in kiwifruit.

Development of long-read sequencing technologies, such as the Pacific Biosciences (PacBio) HiFi and Oxford NanoporeTechnology (ONT) ultra-long platforms, enables the leveraging of extremely long reads for resolution of the most complex structures and assembly of the highly repetitive regions on genomechromosomes [8]. Meanwhile, multiple assemblers developed using different algorithms have offered us an opportunity to create the best possible assembly and even achieve the telomere-to-telomere (T2T) and gap-free genome. In recent years, based on the integration of two cutting-edge sequencing technologies and multiple assembly strategies, complete T2T and gap-free genomes have been obtained in several model plants, such as *Arabidopsis* [8, 9], rice [10], and banana [11]. These T2T and gap-free genome assemblies are necessary to ensure that all genomic variants are discovered and studied.

In this study, we have incorporated datasets of PacBio HiFi reads, ONT ultra-long reads, and Hi-C reads to successfully bridge all the remaining assembly gaps across each chromosome of *A. chinensis* cv. ‘Hongyang’ (2*n* = 2*x* = 58), an elite variety widely cultivated in China. These efforts resulted in the first T2T and gap-free kiwifruit reference genome publicly available to date, with *de novo* assembly of two haploid-resolved haplotypes, named HY4P and HY4A. This T2T genome assembly (hereafter named Hongyang v4.0) reveals the structural characteristics of highly repetitive regions such as telomeres and centromeres for the first time, laying a valuable foundation for a new understanding of the whole structure and function of the kiwifruit genome.

## Results

### Assembly of highly contiguous and phased diploid genome

The continuity of genome assembly is mainly dependent on the sequence depth, length, and quality of the raw reads. To achieve a T2T kiwifruit genome assembly, multiple sequencing platforms were applied for *de novo* genome sequencing of *A. chinensis* ‘Hongyang’, whose genome has already been sequenced and assembled twice, accompanied by upgraded annotations [2, 4, 12]. In total, we generated 31.9 Gb (~52× coverage) of PacBio HiFi reads and 19.0 Gb (~31× coverage) of ONT ultra-long reads by using the PacBio Sequel II and ONT platform (Supplementary Data Table S1). The N50 length of HiFi reads was >14.3 kb, whereas the N50 length of ONT reads reached up to 100.9 kb (Supplementary Data Fig. S1 and Supplementary Data Table S1). Additionally, chromosome conformation capture sequencing (Hi-C) libraries were sequenced by paired-end technology, producing 93.1 Gb (~150× coverage) clean reads for the downstream grouping, ordering, orientation, and verification of assembled contigs (Supplementary Data Table S1).

Next, different assembly strategies were employed to construct the T2T and gap-free kiwifruit genome (Supplementary Data Fig. S2). Depending on comprehensive comparisons, we developed a strategy that allows using PacBio HiFi reads to assemble the chromosome-scale genome coupled with ONT ultra-long reads to fill the gaps. Based on HiFi reads, hifiasm [13] was adopted to construct the phased diploid assembly, producing 616.2 Mb primary contigs and 604.0 Mb alternate contigs with N50 values of 19.0 and 18.0 Mb in length, respectively (Table 1). With the aid of Hi-C reads, the assembled HiFi contigs were further grouped, ordered, and orientated into pseudochromosomes (hereafter named Hi-C pseudochromosomes) by the combined use of Juicer [14] and 3D-DNA [15]. Although Hi-C data could facilitate the scaffolding of contigs, it may also bring new gaps and incorrect joins into the pseudochromosomes by optionally splitting the contigs in those regions lacking Hi-C coverages. Thus, we re-mapped the HiFi contigs against the Hi-C pseudochromosomes for *de novo* genome assembly with a reference-guided strategy by using a custom Perl script (https://github.com/aaranyue/CTGA) and subsequently obtained their new pseudochromosomes (hereafter named Ref-guided pseudochromosomes) with only 9 and 16 gaps scattered within the primary and alternate haplotypes, respectively (Supplementary Data Figs S3 and S4 and Supplementary Data Tables S2 and S3). Meanwhile, the ONT ultra-long reads were separately assembled by Canu [16] and NextDenovo (https://github.com/Nextomics/NextDenovo), and further polished by Pilon [17] based on the HiFi reads. Then, the resulting ONT contigs were used to fill any unclosed gaps in the Ref-guided pseudochromosomes also using the custom Perl script (https://github.com/aaranyue/CTGA). As a result, all the 25 gaps (9 in the primary haplotype and 16 in the alternate haplotype) were successfully filled with bridged ONT contigs (Supplementary Data Figs S5 and S6). Finally, we achieved the chromosomal-level and gap-free reference genome, which was named Hongyang v4.0 in the wake of the recently released genome version of Hongyang v3.0 [4]. Meanwhile, their chromosome identifiers were assigned by referring to Hongyang v1.0 [2] and v2.0 [12]. Whole-genome comparisons showed a high degree of synteny between Hongyang v4.0 and the two previously released genome versions, Hongyang v2.0 [12] and v3.0 [4], indicating the high quality of the genome assembly obtained in this study (Supplementary Data Fig. S7).

**Table 1:** Summary statistics of kiwifruit genome assemblies.

**Genomic feature**	**HY4P**	**HY4A**
Total size of assembled contigs (Mb)	616.2	604.0
Number of contigs	267	208
N50 value of contig length (Mb)	19.0	18.0
Total size of assembled genomes (Mb)	606.1	599.6
Total size of unanchored contigs (Mb)	10.8	9.7
Number of base chromosomes	29	29
Number of gap-free chromosomes	29	29
Number of telomeres (pairs)	57 (28)	58 (29)
Number of definite centromeres	29	29
TE size (%)	42.34	41.34
GC content (%)	35.42	35.41
Genome BUSCOs (%)	99.3	99.3
LTR assembly index score	16.38	15.98
Number of genes/transcripts	45 809/51 252	45 434/51 215
Number of shared genes	37 648	37 579
Number of specific genes	8161	7855
Gene BUSCOs (%)	96.9	96.8

**Figure 1:**
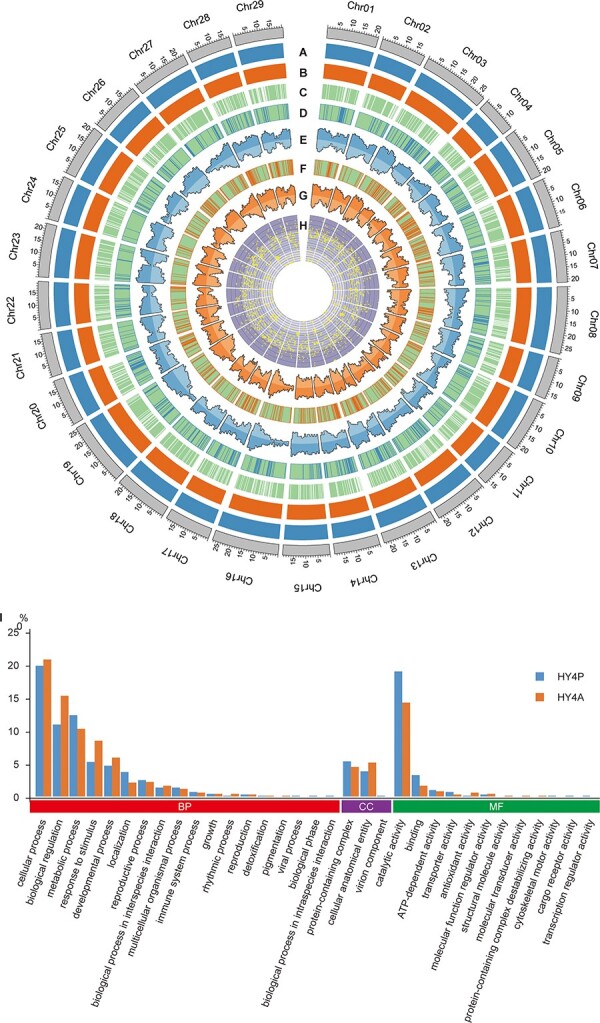
Gap-free and haploid-resolved assembly of Hongyang v4.0. (A) Histogram of the primary haplotype HY4P is shown as blue bar graphs. (B) Histogram of the alternate haplotype HY4A shown as orange bar graphs. (C) Histogram of the genome assembly Hongyang v3.0 shown as green bar graphs, with the remaining gaps labeled as white blocks (D) Presence/absence variations between HY4P and Hongyang v3.0 labeled as blue blocks. (E) Genome-wide counts of protein-coding genes (outside tracks) and high expression genes (inside tracks) in 100-kb bins of HY4P. (F) Presence/absence variations between HY4A and Hongyang v3.0 labeled as orange blocks. (G) Genome-wide counts of protein-coding genes (outside tracks) and high expression genes (inside tracks) in 100-kb bins of HY4A. (H) Global distribution of SNP density between HY4P and HY4A with the display mode of larger values outside and smaller values inside. Ranges of different values are marked with different shapes: triangles (large values), circles (medium values), and squares (small values). This figure was generated by Circos software. (I) New-found genes predicted in the extra sequence segments of Hongyang v4.0 are enriched and assigned to biological process (BP), cellular component (CC), and molecular function (MF) categories according to GO annotation.

Hongyang v4.0 possesses two haplotype-resolved haplotypes, the primary haplotype (hereafter named HY4P) and the alternate haplotype (hereafter named HY4A), containing 29 pseudomolecules with a total length of 606 055 014 and 599 569 706 bp, respectively (Table 1). Compared with Hongyang v3.0 [4], HY4P and HY4A include ~16.4–22.9 Mb of added sequence sizes with a definite improvement of contiguity throughout both haplotypes. Remarkably, we have achieved the gap-free reference genome assembly across each haplotype of Hongyang v4.0 by completely filling all the 646 remaining gaps in Hongyang v3.0 (Fig. 1A–C and Table 1). Furthermore, Hongyang v4.0 allows correction of the previously misoriented or misassembled regions with assistance from the preliminary HiFi contigs that are long enough to span the ambiguous regions (Supplementary Data Table S4). As a result, we acquired 5061 extra sequence segments (~39.5 Mb in total) in HY4P and 5124 extra sequence segments (~38.6 Mb in total) in HY4A, which are likely to affect 4263 and 4244 protein-coding genes of each assembly, accounting for ~9.31% and ~ 9.34% of the total genes (45 809 in HY4P and 45 434 in HY4A), respectively (Fig. 1D–G and Supplementary Data Tables S5 and S6).

Functional enrichment analysis based on Gene Ontology (GO) annotation revealed that the new-found genes predicted in the extra sequence segments of Hongyang v4.0 tend to possess ‘cellular process’ (in ‘biological process’), ‘protein-containing complex’ (in ‘cellular component’) and ‘catalytic activity’ (in ‘molecular function’) categories in both HY4P and HY4A as summarized at level 2, expanding our knowledge of cell communication and metabolic network architecture based on protein–protein interactions in kiwifruit (Fig. 1I). Among them, a certain number of cellular activities were associated with plant resistance to pathogen infection. The enriched GO terms included ‘plant-type hypersensitive response’ (GO:0009626, *P*-value ≅ 0), ‘defense response to other organism’ (GO:0098542, *P*-value <.05) and ‘regulation of immune response’ (GO:0050776, *P*-value <.05) (Supplementary Data Tables S7 and S8). In addition, several biosynthetic pathways were also found to be significantly enriched in these new-found genes, such as ‘*O*-malonyltransferase activity’ (GO:0050736, *P*-value <.05), ‘flavonol-3-*O*-beta-glucoside *O*-malonyltransferase activity’ (GO:0047165, *P*-value <.05), ‘anthocyanin 5-aromatic acyltransferase activity’ (GO:0047183, *P*-value <.05) and ‘anthocyanin 5-*O*-glucoside 6′-*O*-malonyltransferase activity’ (GO:0033810, *P*-value <.05), whose products may contribute to aromas and colors of the fruits (Supplementary Data Tables S7 and S8).

A comparison between the two haplotypes of Hongyang v4.0, HY4P and HY4A, showed that they contain a set of similar genomic features, including closed genome sizes, parallel repeat contents, and similar gene numbers (Table 1 and Supplementary Data Table S9). The whole-genome alignments further revealed a large scale of conserved synteny in both haplotypes (Supplementary Data Fig. S8). Meanwhile, 3 950 488 single-nucleotide polymorphisms (SNPs), 405 133 insertions, 402 879 deletions, 90 inversions, 1605 translocations, and 6120 duplications were detected, constituting a substantial source of genetic variation between the two haplotypes (Fig. 1H and Supplementary Data Table S10). In total, these variations spanned 41.4 and 33.1 Mb, representing 6.83% and 5.52% of the genome content of the two haplotypes, respectively. Among these variants, ~1.62% of SNPs and 0.76% of small insertions and deletions (InDels) caused changes of start/stop codons, splicing sites, encoded amino acids, or frameshifts, which may contribute to the diversity of gene functions in kiwifruit (Supplementary Data Table S11). Furthermore, 75 227 shared genes (37 648 from HY4P and 37 579 from HY4A) belonging to 28 837 orthologous gene families were obtained from HY4P and HY4A, representing a core set of gene clusters in Hongyang v4.0 (Table 1 and Supplementary Data Table S12). On the other hand, 8161 and 7855 genes were specific to HY4P and HY4A, respectively, suggesting their independent evolution after parental divergence of kiwifruit (Table 1).

### Assessment of the HY4P or HY4A haplotypes of Hongyang v4.0

**Figure 2:**
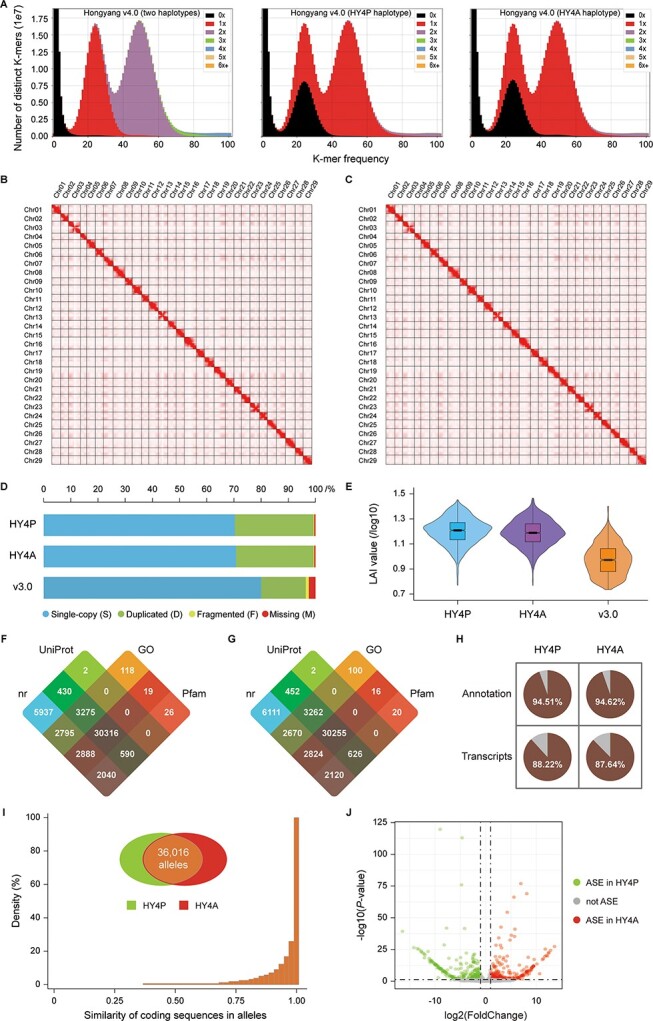
Quality assessment of the genome assembly. (A) *K*-mer analysis shows comparisons of HiFi reads to Hongyang v4.0 with KAT. The plots are colored to illustrate how many times specific *K*-mers from the reads appear in the assembly. Black represents *K*-mers missing from the assembly, while red, purple, green, blue, yellow, and orange represent *K*-mers that appear once, twice, three times, four times, five times, and six or more times in the assembly, respectively. (B) Heat map displaying Hi-C interactions of HY4P pseudomolecules. (C) Heat map displaying Hi-C interactions of HY4A pseudomolecules. (D) BUSCO assessments exhibiting proportions classified as Complete and single-copy (S, blue), Complete and duplicated (D, green), Fragmented (F, yellow), and Missing (M, red) categories. (E) Comparison of LAI scores among the three assemblies of HY4P, HY4A, and Hongyang v3.0. (F) Numbers of HY4P protein-coding genes annotated in the NCBI nr plant, UniProt, GO and Pfam databases are illustrated by a Venn diagram. (G) Numbers of HY4A protein-coding genes annotated in the NCBI nr plant, UniProt, GO and Pfam databases are illustrated by a Venn diagram. (H) Proportions of genes that could be functionally annotated and transcriptionally detected in HY4P and HY4A. (I) Statistics of sequence identities between the two genes in each allele. The Venn diagram in the upper middle denotes the number of alleles found within genome-wide alignment blocks. (J) Identification of ASE genes between haplotypes HY4P and HY4A. Green and red dots indicate ASE genes with biased expression toward HY4P and HY4A, respectively, whereas gray dots represent genes that are not ASE.

Although Hongyang v4.0 was a diploid genome assembly, both haplotypes had higher completeness and contiguity than the assembly of Hongyang v3.0. For a more comprehensive sense, multiple methods were further performed to evaluate the accuracy of HY4P and HY4A. First, a perfect spectra graph plotted by the KAT program [18] clearly demonstrated that the phasing of the assembled haplotypes is correct for both HY4P and HY4A of Hongyang v4.0 (Fig. 2A). Second, chromosome conformation capture sequencing (Hi-C) data visualized by Juicebox [19] showed a high consistency across all chromosomes of HY4P and HY4A, proving their accuracy of ordering and orientation (Fig. 2B and C). Third, genome completeness was evaluated by high mapping rates with various raw sequences, such as raw PacBio HiFi reads, ONT ultra-long reads, and Hi-C short reads, all of which mapped at >99% across each assembly (Supplementary Data Table S13). Additionally, the mapping rates of 36 public RNA-sequencing (RNA-seq) datasets against Hongyang v4.0 were all greater than the mapping rates of the same data against Hongyang v3.0 (Supplementary Data Table S14). Subsequently, quality assessment with the BUSCO tool [20] revealed that complete sequences of HY4P and HY4A accounted for 99.3% (1142 single-copy and 460 duplicated genes out of 1614) and 99.3% (1135 single-copy and 467 duplicated genes out of 1614) of the conserved core eukaryotic gene set, respectively, whereas the score value of complete sequences was 96.4% for Hongyang v3.0 (Fig. 2D and Table 1). Finally, long terminal repeat (LTR) annotation [21] showed that the LTR assembly index (LAI) values for HY4P and HY4A were 16.38 and 15.98, respectively, both of which met the standard of reference genomes and were significantly higher than Hongyang v3.0 (~10.63) (Fig. 2E and Table 1).

For gene content assessment, a total of 45 809 and 45 434 protein-coding genes were respectively identified in HY4P and HY4A, capturing 96.9% and 96.8% of a BUSCO 1614 reference gene set (Table 1). Meanwhile, 51 252 and 51 215 transcripts were predicted with an average of 1.12 and 1.13 splice variants from the entire genes of HY4P and HY4A (Table 1). Among them, 48 436 (94.51%) and 48 458 (94.62%) could be functionally annotated to a suite of comprehensive databases (Fig. 2F–H), whereas 45 216 (88.22%) and 44 886 (87.64%) could be transcriptionally detected by the 36 public RNA-seq datasets (Fig. 2H). Based on our phased haplotypes, it would be possible to investigate gene sequence divergence and the expression imbalance of each allele without resequencing the parental genomes. Consequently, a total of 36 016 alleles were found within all the 5464 alignment blocks, sharing an average of 97.27% protein-coding sequence identity between HY4P and HY4A (Fig. 2I and Supplementary Data Table S15). Among them, 21.14% of alleles (7615 out of 36 016) exhibited significant allele-specific expression (ASE) (*P*-value <.05 and false discovery rate, FDR < .05) across diverse kiwifruit tissues, including fruit, phloem and root employed in the 36 RNA-seq datasets (Fig. 2J and Supplementary Data Tables S14 and S15). GO enrichment analysis showed that these ASE genes are significantly enriched in multiple biological processes and molecular functions, such as ‘alkaloid metabolic process’ (GO:0009820, *P*-value <.05), ‘flavonol synthase activity’ (GO:0045431, *P*-value <.05) and ‘cellular glucan metabolic process’ (GO:0006073, *P*-value <.05) (Supplementary Data Table S16). Sequence alignments suggested that 430 and 706 ASE genes are most probably caused by genetic variations of SNPs and InDels in the upstream regions (Supplementary Data Tables S17 and S18).

To retain uniform criteria during comparison across different genome assemblies, we then annotated the Hongyang v3.0 assembly also by BRAKER [22] with the same evidence data used for Hongyang v4.0 annotation, and obtained 46 395 putative protein-coding genes with a BUSCO score of 89.3% the same as the score of 89.1% reported for Hongyang v3.0). Notably, the BUSCO score of Hongyang v3.0 was much lower than that of Hongyang v4.0, although they contained the same size of protein-coding genes in total. So, we manually checked the list of missing BUSCOs in Hongyang v3.0 and the complete BUSCOs in Hongyang v4.0, and then found the specific genes within the complete category of Hongyang v4.0 (Supplementary Data Table S19). Subsequently, homologous genes in Hongyang v3.0 were identified by blasting the specific genes in Hongyang v4.0 against the whole genome of Hongyang v3.0. Sequence comparisons showed that Hongyang v3.0 contained a large number of truncated genes resulting from frameshift mutations due to deletions or insertions of nucleotide bases in numbers that are not multiples of three (Supplementary Data Fig. S9). Moreover, we performed genome-wide analysis of the nucleotide-binding site and leucine-rich repeat receptor (*NLR*) genes, which constitute the largest group of plant disease resistance (*R*) genes. As a result, a total of 219, 212, and 151 *NLR* genes were respectively annotated in HY4P, HY4A and Hongyang v3.0, accounting for 0.48%, 0.47%, and 0.33% of the coding genes (Supplementary Data Table S20). Apparently, the number of *NLR* genes identified in both HY4P and HY4A is much larger than that in Hongyang v3.0, further illustrating that Hongyang v4.0 will be highly useful for functional gene mining and molecular breeding in kiwifruit.

### Detection of telomere and centromere locations in chromosomes

**Figure 3:**
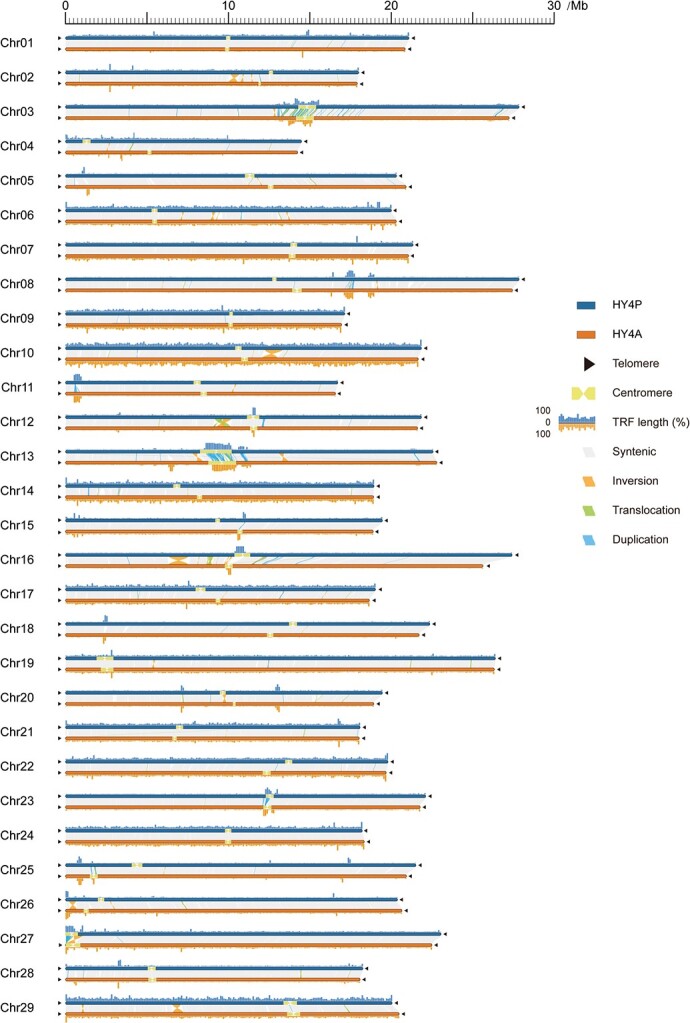
Structure of T2T and gap-free chromosomes in Hongyang v4.0. All 29 chromosomes of HY4P and HY4A are drawn to scale and the ruler indicates chromosome length. Black triangles indicate the presence of telomere sequence repeats. Yellow dumbbell shapes represent the locations and sizes of centromeric regions. TRF percentage distribution is plotted above or under their respective chromosomes in 100-kb bins. Collinearity analysis between HY4P and HY4A with syntenic regions are shown as gray lines, inversions as orange lines, translocations as green lines, and duplications as blue lines.

The telomere is a region of highly repetitive DNA sequences that resides at the end of the chromosome, which could protect the ends of chromosomes from becoming frayed or tangled [23]. In plants, the telomere sequences are extremely conserved in unique repeat 7-bp nucleotide units (CCCATTT at the 5′ end and TTTAGGG at the 3′ end) [24]. Using the normalized and unified sequence AAACCCT as query, we detected 57 and 58 distinct telomeres that resulted in 28 and 29 intact chromosomes at a T2T level in the HY4P and HY4A haplotypes of Hongyang v4.0, respectively (Fig. 3 and Table 1). The length of identified telomeres ranged from 1946 to 14 126 nucleotides in HY4P and 497 to 16 387 nucleotides in HY4A, with the longest one located at the left end of Chr26 in HY4A (Supplementary Data Tables S21 and S22). Comparatively, we only detected one telomere repeat unit at the left end of Chr27 in HY4P, resulting in the solely missing telomere identified in Hongyang v4.0 (Fig. 3 and Supplementary Data Table S21).

Centromeres are essential for the maintenance of chromosome integrity and the accuracy of chromosome segregation during cell division. Although they are also composed of tandem repeat sequences, the repeat monomers and chromosome locations display great variations between individuals, even between tissue and cell types [25]. Till now, little has been known about the sequence and structure of centromeres in kiwifruit. Using the Tandem Repeats Finder (TRF) tool [26], we first identified whole-genome tandem repeats in our genome assembly. Due to the requirement to bind centromere-specific histones, centromeric repeat monomers are commonly 100–200 bp long [27]. Thus, we extracted and retained only the repeat monomers with length ranging from 100 to 200 bp. Subsequently, CD-HIT [28] was used for clustering these monomers to reduce sequence redundancy and improve the precision of centromere localization based on a sequence similarity search. In total, 22 574 representative monomers were obtained from all the clusters and then applied for whole-genome alignment to gain the records of aligned locations on each reference chromosome. Finally, the continuous and high-frequency regions were proposed to be approximate centromeric sequences. Meanwhile, the boundaries of each centromere could be delimited, with the sizes varying from 111 852 to 2 091 436 bp in HY4P and from 101 559 to 1 851 899 bp in HY4A (Fig. 3 and Supplementary Data Tables S23 and S24). In total, there were 422 and 375 protein-coding genes located within the chromosome centromeres of HY4P and HY4A, respectively (Supplementary Data Tables S23 and S24). Function enrichment analysis showed that these genes were significantly enriched in multiple cellular components, including ‘cortical actin cytoskeleton’ (GO:0030864, *P*-value <.05), ‘actin cap’ (GO:0030478, *P*-value <.05), and ‘retromer, tubulation complex’ (GO:0030905, *P*-value <.05), suggesting their potential functions in the segregation of homologous chromosomes during cell division (Supplementary Data Tables S25 and S26).

After estimating the centromeres, we re-used the TRF tool [26] to dissect their repeat monomers. Typically, the identified kiwifruit centromeres represented a particularly complex region with a set of monomers repeated and nested in tandem (Supplementary Data Tables S27 and S28). When more repeat monomers were found within one centromere, only the monomer occupying the majority was regarded as a centromeric monomer. As a result, the repeat monomers of 29 centromeric sequences were all determined in each haplotype, with the length ranging from 152 to 195 bp in HY4P and from 152 to 193 bp in HY4A, among which the 153-bp length is the most frequent and considered as the representative length of centromeric monomers in kiwifruit, termed *Ach-CEN153*. Sequence comparison showed that *Ach-CEN153* had 32.8%–52.1% similarity with the *CEN180* monomer in *Arabidopsis* [8] and 25.8%–48.0% similarity to the *CentO* monomer in rice [10].

### Inspection of the identified centromere and monomer in kiwifruit

**Figure 4:**
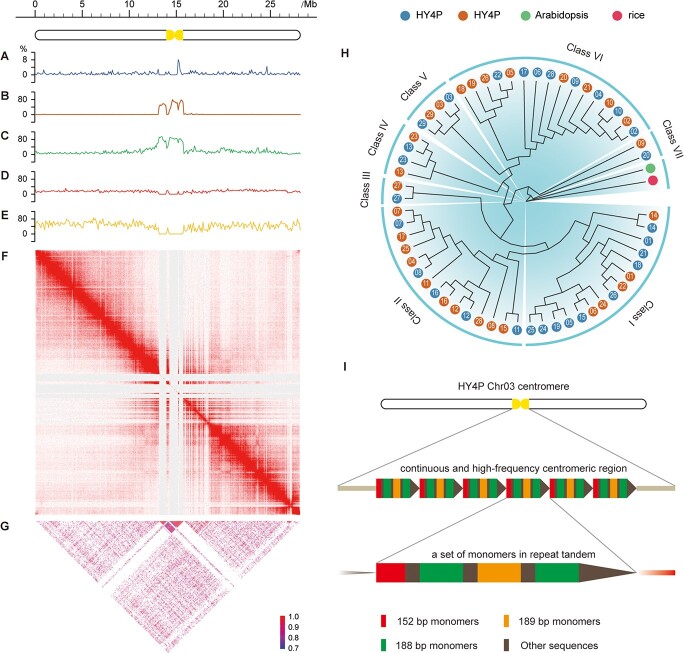
Characterization of the TRF-defined centromere on Chr03 of HY4P. The schematic diagram shows the complete chromosome (Chr03) at the top left; the centromere is labeled as a yellow dumbbell shape. (A–E) Density and distribution of (A) TRFs, (B) BLAST results, (C) class I retrotransposons, (D) class II DNA transposons, and (E) protein-coding genes across the whole chromosome (Chr03) in 100-kb bins. (F) Heat map showing Hi-C interactions of the chromosome (Chr03). (G) Heat map showing pairwise similarity of a 50-kb sequence along the whole chromosome (Chr03). (H) NJ phylogenetic tree of 58 centromeric monomers constructed from the two haplotypes of Hongyang v4.0. Seven branches are grouped according to sequence similarity. *Arabidopsis CEN180* and rice *CentO* are used as outgroups. Numeric values within each circle indicate the serial numbers of chromosomes. (I) Centromeres composed of more than one monomer. As shown on Chr03 of HY4P, a set of three monomers with different length are organized in a repeat unit, which could reiterate hundreds to thousands of times in tandem head-to-tail fashion to form the continuous and high-frequency centromeric region. The monomeric sequences differ by as much as 50%, specifically as follows: 37.6% between 152- and 188-bp monomers, 52.6% between 152- and 189-bp monomers, and 41.2% between 188- and 189-bp monomers.

In support of the centromeres defined by the TRF tool (hereafter named TRF-defined centromeres) (Fig. 4A), we adopted a variety of methods to validate the centromeric localization on each chromosome of HY4P and HY4A. First, our strategy for sequence similarity search using the BLAST program was able to dramatically enhance visibility in the target centromeric regions (Fig. 4B). Second, the distribution of LTR repeats revealed that class I retrotransposons are more common within or in the vicinity of the identified TRF-defined centromeres (Fig. 4C), while class II DNA transposons are more evenly distributed across the whole chromosome (Fig. 4D). By analyzing the density of LTR repeats, we showed that a small proportion (~2%) of the centromere on each chromosome carried a very large percentage of retrotransposons (~60%), but a relatively small percentage of DNA transposons (~10%), suggesting a strong relationship between retrotransposons and centromeres (Supplementary Data Tables S29 and S30). Third, the TRF-defined centromeres were detected with a low density of protein-coding genes (Fig. 4E). Fourth, the regions of TRF-defined centromeres had approximately the same coordinates as the strong interaction signals in the genome-wide Hi-C contact maps (Fig. 4F). Finally, we drew a heat map showing pairwise similarity of a 50-kb sequence from one terminal end to another of each chromosome, and observed that the TRF-defined centromeres had the highest similarity over wide scale ranges on the chromosomes (Fig. 4G).

After confirming centromere localizations, we further conducted a genome-wide characterization of the centromeric monomers. Based on the monomers from 29 chromosomes of two haplotypes, HY4P and HY4A, a phylogenetic tree was constructed using *Arabidopsis CEN180* [8] and rice *CentO* [10] as outgroups. Our results showed that all kiwifruit monomers were separated from *CEN180* and *CentO*, and could be subdivided into seven distinct branches (Fig. 4H). Within the same branches, monomers from 15 homologous chromosomes of the two haplotypes were clustered together (Supplementary Data Table S31), revealing that the similarity of monomers on homologous chromosomes is higher than the similarity across chromosomes, supporting models of repeated amplification events involving the central domain and local homogenization [10, 29]. Additionally, those centromeric monomers from homologous chromosomes dispersed in different branches might suggest a rapid evolution after hybridization of the two parents of the ‘Hongyang’ cultivar. In fact, most of the centromeres contain more than one monomer, explaining why centromeric sequences are not broadly conserved throughout the evolutionary process. As shown on Chr03 of HY4P, the centromere contained three types of monomers in the form of tandem repeats, differing by up to 50% (Fig. 4I).

## Discussion

Kiwifruit is a dioecious plant setting delicious fruits containing exceptionally nutritional metabolites. Nowadays, hundreds of cultivars are grown worldwide with the majority bred from *A. chinensis*. A reference genome is indispensable for trait discovery and genetic improvement. Since 2013, a total of three genome versions of *A. chinensis* ‘Hongyang’ have been released and updated, constituting a valuable resource for the scientific community in kiwifruit [2, 4, 12]. However, dozens of megabases embedded in the individual genome sequences remained unassembled or unplaced. In the present study, we describe a fourth *de novo* genome assembly of *A. chinensis* ‘Hongyang’ (Hongyang v4.0) and successfully achieve the first T2T and gap-free kiwifruit genome by incorporating two state-of-the-art sequencing technologies and multiple genome assembly strategies. Hongyang v4.0 represents higher sequence contiguity and assembly quality than all of the three previously released versions [2, 4, 12]. Even compared with the latest version, Hongyang v3.0 [4], we have identified up to 4200 new-found protein-coding genes from ~39 Mb extra chromosome sequences in the current assembly, which might exert an overall impact on plant growth and development, crop yields and disease resistance of kiwifruit. For instance, a considerable number of genes involved in the regulation of the immune response were functionally enriched and the ortholog of one member (HY4P Gene ID: Achv4p22g034066) in *Arabidopsis thaliana* encodes a resistance (*R*) gene (TAIR Gene ID: At1g58602) that participates in the induction of protein ubiquitination during bacterial infection [30]. Meanwhile, the available T2T and gap-free genome made it possible to study the structure, function, and evolution of centromeres in kiwifruit for the first time. Our assembly showed extensive variations in size and location, but relative similarities in structure and sequence of the centromeres, demonstrating an adaptive process of local expansion and homogenization in kiwifruit, which is consistent with the model presented in the analyses of rice centromeres [10]. Further, the large number of actin-related genes identified from centromeric sequences demonstrated that chromosome centromeres not only play physical roles for linking pairs of sister chromatids, but also have genetic features to participate in the regulation of cell division.

During the assembling process, high-sequencing-depth long reads generated by PacBio HiFi and the ONT platform actually complement each other due to their relative advantages, offering a strong possibility of improving genome reconstruction with multiple trials [31]. Our use of high-fidelity PacBio HiFi reads (N50 length of ~14.3 kb) led to a clear improvement in the sequence continuity of contigs, which could fill the majority of gaps and correct extensive misassemblies in the previous three versions. Furthermore, we introduced ONT ultra-long reads (N50 length of >100 kb) to fill the remaining gaps that HiFi reads could not span. This helped us to achieve the completely gap-free kiwifruit reference genome Hongyang v4.0. Based on our results, it is recommended to use the HiFi contigs assembled by hifiasm [13] as a fixed backbone and then perform manual handling with the ONT contigs to fill the small amount of remnant gaps, which is identical to the genome assembly strategies adopted in rice, watermelon, and barley [10, 32, 33], but slightly different from those in *Arabidopsis* and banana [8, 11]. Comprehensively, the choice of genome assembly strategy should depend on practical testing effects, which may vary among different species. Additionally, the sequencing depths of PacBio HiFi reads and ONT ultra-long reads could also have an appreciable influence on the strategy adopted. At present, it is still difficult to fully assemble a high-quality T2T and gap-free genome with any one sequencing platform alone. The combination of HiFi reads and ONT reads is a highly recommended method, which has produced better results from our studies.

In addition, our scaffolding with Hi-C reads further facilitated the faithful construction of nearly all contigs (~98.3%) to pseudochromosomes. However, if a contig contained any regions lacking Hi-C coverages, it was likely to be split into two or more shorter contigs [34], which would increase the number of contigs and bring new gaps in the final genome assembly. But in fact, those contigs with low-spanning Hi-C alignments often showed better support by HiFi reads. For this conflict, we followed a priority order of HiFi reads, ONT reads, and Hi-C reads during the processing of our genome assembly. Thus, using the Hi-C pseudochromosomes as references, we reconstructed the HiFi contigs with a reference-guided strategy by using a custom Perl script, which was proved to be an effective and efficient strategy in the present study.

Another strength of our adopted strategy is the ability to construct two haplotypes, HY4P and HY4A, in Hongyang v4.0. Kiwifruit plants are functionally dioecious, leading to a relative high level of heterozygosity in the genome. Compared with the three previous genomes using a collapsed assembly method, our haploid-resolved haplotypes could provide more phasing information among heterozygous ranges between homologous chromosomes of the two haplotypes, such as phased SNPs, InDels and structural variations [35]. These allelic variations may have potentially important cellular and biological functions during early domestication and contiguous breeding of kiwifruit. In Hongyang v4.0, the whole-genome comparison between HY4P and HY4A has revealed a total of 41.4 and 33.1 Mb genomic variations in each haplotype, whose detection is of great significance in the field of genetics and genomics. Furthermore, genome phasing can help us to study allele-specific expression and allelic imbalance at the transcriptional level, which has been regarded as an important molecular mechanism for causing heterosis in many crops [36–38]. Our results uncovered a significant enrichment of biosynthetic pathways that are associated with the major characteristic secondary metabolites (e.g. flavonoid, alkaloid, and fructose) in 7615 ASE genes, suggesting a potential dominance effect of heterosis in the diploid kiwifruit genome, partially at least. Thus, the availability of a haploid-resolved genome assembly provides the first opportunity to understand kiwifruit phenotypic trait inheritance and variability in cases of compound heterozygosity, allele-specific expression, or *cis*-regulatory variants.

## Materials and methods

### Plant materials and sample collection

*A. chinensis* cv. ‘Hongyang’, the same individual as that used for the first kiwifruit genome sequencing [2], was used in this study. Green wood cuttings were picked and grown in a greenhouse at Anhui Agricultural University, Anhui Province, China, under 25°C, 12-/12-hour days. Fresh young healthy leaves were collected from 2-week-old branches and separately packaged for PacBio HiFi, ONT ultra-long, and Hi-C sequencing. After collection, these tissues were immediately placed in a cryonic chamber with liquid nitrogen and then preserved at −80°C for further usage.

### Library preparation and DNA sequencing

High-molecular-weight genomic DNAs (gDNAs) were separately extracted from each leaf tissue sample by using a slightly modified cetyltrimethylammonium bromide (CTAB) method [39]. The quality and quantity of the isolated gDNAs were evaluated with an Agilent 2100 Bioanalyzer (Agilent Technologies, CA, USA) and a Qubit fluorometer instrument (Thermo Fisher Scientific, MA, USA), respectively. For PacBio HiFi sequencing, a standard SMRTbell library was prepared with 50 μg of gDNA by using the SMRTbell Express Template Prep Kit 2.0, according to the manufacturer’s instructions. SMRTbell libraries were then sequenced on a PacBio Sequel II system (Pacific Biosciences, CA, USA). For ONT ultra-long sequencing, the gDNAs with larger sizes were selected with Short Read Eliminator XL (Circulomics, MD, USA) following the protocol provided by ONT Community. The library was prepared with the Oxford Nanopore SQK-LSK109 kit according to the manufacturer’s instructions, and then sequenced on a PromethION flow cell. Finally, the Hi-C sequencing library was prepared and sequenced based on a previously published protocol [40].

### Genome assembly and gap filling

As shown in Supplementary Data Fig. S2, a cluster of bioinformatics tools based on different algorithms were employed to *de novo* assemble the T2T and gap-free genome of *A. chinensis* ‘Hongyang’. First, the raw data produced by the PacBio Sequel II system were processed through the SMRT Analysis software suite (version 5.1.0; https://www.pacb.com/products-and-services/analytical-software/smrt-analysis/), whereas the consensus HiFi reads were produced by the CCS subprogram (https://github.com/PacificBiosciences/ccs) with default parameters. Subsequently, the obtained highly accurate HiFi reads were *de novo* assembled using phased assembly graphs with hifiasm v0.16.1 [13], hereafter named HiFi contigs. Then, the two sets of HiFi contigs (including primary and alternate contigs) were validated, grouped, sorted, and anchored with the Hi-C reads to generate two pseudochromosomes, hereafter named Hi-C pseudochromosomes, by using Juicer [14] and 3D-DNA [15] in turn. As certain contigs have been optionally split in regions that were classified as invalid interaction pairs due to the lack of Hi-C coverages, new gaps and incorrect joins would be introduced into the Hi-C pseudochromosomes. After manual examination with the Integrative Genomics Viewer (IGV) tool [41], the breakpoint locations caused by Hi-C analysis could be inferred from the HiFi reads with obvious extents and sufficient coverages. Thus, we tended to completely retain the HiFi contigs assembled by hifiasm. To achieve this, we have developed a custom Perl script (https://github.com/aaranyue/CTGA) to re-map the HiFi contigs against the Hi-C pseudochromosomes by a reference-guided strategy. The CTGA script enables the use of Hi-C pseudochromosomes to guild the ordering and orienting of HiFi contigs, but without splitting them. Finally, the new pseudochromosomes were obtained and hereafter named Ref-guided pseudochromosomes.

In addition, the ONT ultra-long reads were separately performed for *de novo* assembly by the Canu v2.1.1 [16] and NextDenovo v2.4.0 (https://github.com/Nextomics/NextDenovo), and further polished by Pilon [17] based on the HiFi reads with default parameters. Then, the generated contigs, hereafter named ONT polished contigs, were used to fill gaps in the Ref-guided pseudochromosomes by our CTGA script (https://github.com/aaranyue/CTGA). CTGA employed Minimap2 [42] to search the overlapping sequences across gaps with the parameters of alignment length ≥ 1000 bp and percent identity ≥ 80% at both sides. If found, the most consensus sequence was applied to replace the corresponding gap-tied sequence.

### Assembly validation and quality assessment

The haploid-resolved haplotypes were validated by the KAT program [18] with default parameters and a perfect spectra graph was acquired, which clearly showed that a complete and well-separated assembly of both haplotypes was achieved. The completeness of genome assembly was estimated using BUSCO assessment (version 5.2.1) [20], which contained 1614 genes in the Embryophyta OrthoDB v10 dataset (https://www.orthodb.org). The continuity of genome assembly was evaluated based on the contigs’ N50 values and the LAI scores [21]. The accuracy of genome assembly was evaluated by sequence alignments with multiple types of data as follows. The HiFi and ONT reads were aligned by Minimap2 [42] and the short reads were aligned by BWA v0.7.17 [43], whereas the RNA-seq reads were aligned by Hisat2 v2.1.0 [44] and counted by featureCounts [45]. In addition, we utilized Juicebox [19] to visualize and check the Hi-C data.

### Repeat identification and gene prediction

The transposable elements (TEs) were annotated by using the comprehensive pipeline EDTA [46] with default parameters. The tandem repeats (TRs) were identified by TRF software with parameters (2 7 7 80 10 50 500 -f -d -m). Each genome assembly was hard- and soft-masked by RepeatMasker [47]. Putative genes as well as their protein-coding regions were predicted by a comprehensive Perl pipeline, BRAKER v2.1.6 [22]. A total of 36 RNA-seq datasets (Supplementary Data Table S14) were provided for assisting gene prediction in the present study. Only those genes meeting the requirements of starting with ATG, ending with a stop codon, and containing sequences length than 100 amino acids were reserved. For gene annotation, the BLASTP (version 2.6) and diamond (version 0.9.23) programs were used between the encoded protein sequences and a suite of protein databases, including the NCBI nr plant, Swiss-Prot, TAIR (https://www.arabidopsis.org/), KIR (http://kir.atcgn.com/) and KGD (http://kiwifruitgenome.org/) databases, with an *E*-value threshold of 1*e*−5. Subsequently, the Blast2GO local pipeline (version 3.2) was used to assign GO terms for each protein-coding gene. The motifs and domains within protein sequences were identified with the InterProScan software (version 5.29) by searching against the Pfam database (https://pfam.xfam.org/). The genome-wide nucleotide-binding site and leucine-rich repeat receptor (*NLR*) genes were annotated by the NLR-Annotator tool [48]. The OrthoFinder package (version 2.2.7) [49] was employed to identify gene families between HY4P and HY4A. The enrichment analysis of GO terms was performed using the Hypergenometric test as in our previous description [50].

### Telomere detection and centromere localization

Telomeres were directly detected by searching the normalized and unified sequence AAACCCT within 50 kb of each terminal chromosome sequence. For centromere localization, we first used the TRF tool [26] to identify whole-genome tandem repeats and their monomers. In consideration of the potential to bind centromere-specific histones, only centromeric repeat monomers with length ranging from 100 to 200 bp were retained. Then, CD-HIT [28] was used for clustering these monomers to reduce sequence redundancy and improve the performance of centromere localization. The continuous and high-frequency regions were thought to be approximate centromeric sequences. Meanwhile, the monomer that occupied the majority of each centromere was regarded as the centromeric monomer. After obtaining all the centromeric monomers, their length distribution was measured and the most frequent number was defined as the representative length of kiwifruit centromeric monomers in this study.

### Genome comparison and synteny analysis

Genome-wide comparisons between any two assemblies of HY4P, HY4A, Hongyang v3.0, and Hongyang v2.0 were performed by using the MUMmer (version 4.0.0beta2) toolbox [51] with parameters (--maxmatch -c 500 -b 200 -l 100). Then, the delta filter was used to filter the alignment results with parameters (-m -i 90 -l 100), and show-snps was used to obtain the SNP and InDel information with parameter (-Clr). Finally, mummerplot was employed to generate a dot plot representation of each comparison. Meanwhile, we introduced Synteny and Rearrangement Identifier (SyRI) [52] to identify collinear orthologs, structural variations, and sequence differences by using the alignment results of MUMmer. In addition, a custom Perl script was used to extract information about the location and size of the variations. The effects of genetic variants were predicted by the SnpEff program [53]. Specially, the genomic data of Hongyang v2.0 and v3.0 were downloaded from the KGD database [54].

### Determination of allele-specific expression

As the haplotype-resolved kiwifruit genome assembly is currently available, all alleles can be obtained with only the DNA sequences. First, genome-wide alignment blocks between the two haplotypes of HY4P and HY4A were extracted from the above analysis of synteny. Second, the most similar pairs of genes were identified with the highest sequence similarity of coding proteins between HY4P and HY4A. Here, any gene pairs within alignment blocks were considered as alleles. Then, expression profile analysis of allelic genes was conducted by mapping the 36 public RNA-seq datasets against Hongyang v4.0 using Hisat2 v2.1.0 [44] with only the best match retained for each read. The expected read counts were estimated by featureCounts [45] and differentially expressed allelic genes were identified by DESeq2 [55]. Finally, ASE was determined if the fold change of read counts was no less than 2 with both *P*-value <.05 and FDR <.05. Specifically, these RNA-seq datasets were derived from three tissues (fruit, phloem, and root) with three biological replicates in each experiment (Supplementary Data Table S14).

### Construction of phylogenetic tree

All the sequences of centromeric monomers were aligned by the ClustalW tool (version 2.1) and the maximum likelihood phylogenetic tree was constructed by the MEGA tool (version 10) using the neighbor-joining (NJ) method. The bootstrap process was replicated 1000 times. The monomer sequences of *Arabidopsis CEN180* [8] and rice *CentO* [10] were used as outgroups in our evolutionary tree. Their pairwise sequence alignments were performed by using the EMBOSS Needle program (v6.6.0.0) with parameters (-gapopen 10 -gapextend 0.5).

### Visualization of statistics data

Generally, we used the R language to analyze and graph most of our statistics data. In particular, the ‘barplot’ package was used to display histograms, the ‘ggplots’ package was used to display pie charts and violin plots, the ‘VennDiagram’ package was used to display Venn diagrams, the ‘phyper’ package was used to analyze hypergeometric distribution, the ‘DESeq2’ package was used to determine ASE genes, the ‘ggrepel’ package was used to display volcano plots, and the ‘ggcorrplot’ package was used to display heat maps of sequence similarity.

## Acknowledgements

This work was supported by funds from the National Natural Science Foundation of China (31972474, 90717110) and Hubei Natural Science Fund for Distinguished Young Scholars (2020CFA062).

## Author contributions

Y. Liu and J.Y. conceived the ideas for this paper. Y. Liu, Y. Wu, and H.X. prepared the materials. J.Y., Q.C., Y. Z. Wang, Y. Lin, H.Q., Y. L. Wang, and S.Z. analyzed the data and drew the figures. X.W., S.C., and W.H. provided good guidance for data analysis. J.Y., Y.Y., and C.Y. designed the web server. J.Y., Y. Liu, and Y. Wang wrote the paper. L.Z. and S.W. edited the paper. All authors read and approved the final manuscript.

## Data availability

The raw reads generated in this study have been deposited in the NCBI sequence read archive (SRA) with the accession number PRJNA869178 (http://www.ncbi.nlm.nih.gov/bioproject/PRJNA869178). The custom Perl scripts are all publicly available on GitHub (https://github.com/aaranyue/).

## Conflict of interest

The authors declare that they have no competing interests for this research.

## Supplementary data

Supplementary data are available at *Horticulture Research* online.

## Supplementary Material

Web_Material_uhac264
